# Phylogenetic diversity and molecular evolution of Hantaan virus harbored by *Apodemus chejuensis* on Jeju Island, Republic of Korea, 2022–2023

**DOI:** 10.1371/journal.pntd.0013459

**Published:** 2025-08-19

**Authors:** Juyoung Noh, Kyungmin Park, Seong-Gyu Kim, Ye-rin Seo, Jongwoo Kim, Hee-Kyung Cho, Won-Keun Kim, Jin-Won Song

**Affiliations:** 1 Department of Microbiology, Korea University College of Medicine, Seoul, Republic of Korea; 2 Institute for Viral Diseases, Korea University College of Medicine, Seoul, Republic of Korea; 3 BK21 Graduate Program, Department of Biomedical Sciences, Korea University College of Medicine, Seoul, Republic of Korea; 4 Department of Microbiology, College of Medicine, Hallym University, Chuncheon, Republic of Korea; 5 Institute of Medical Research, College of Medicine, Hallym University, Chuncheon, Republic of Korea; Washington State University, UNITED STATES OF AMERICA

## Abstract

**Background:**

Hantaan virus (HTNV), hosted by *Apodemus* spp., is a well-recognized causative agent of hemorrhagic fever with renal syndrome (HFRS) and poses a crucial global public health concern. Based on the current evidence, HTNV carried by *A. chejuensis* is proposed as the likely etiological agent of HFRS on Jeju Island, Republic of Korea (ROK).

**Methodology/Principal findings:**

In this study, 50 small mammals were collected from five locations in Seogwipo-si and Jeju-si on Jeju Island, ROK, during 2022–2023. Serological and molecular analyses revealed HTNV prevalence rates of 34% (16/47) and 27.7% (13/47), respectively. Using a multiplex polymerase chain reaction-based nanopore sequencing approach, nine complete HTNV genomes were sequenced from the lung tissues of *A. chejuensis*, representing the first comprehensive genomic characterization of HTNV from Seogwipo-si (Hogeun-dong) and Jeju-si (Sangdae-ri). Phylodynamic analyses suggest evolutionary divergence and phylogeographic diversity, with four unique amino acid substitutions identified in HTNV genomes from Seogwipo-si.

**Conclusion/Significance:**

This study provides important insights into the genomic surveillance, genetic diversity, and evolutionary dynamics of orthohantaviruses, which are essential for guiding effective public health strategies to control and prevent future HFRS outbreaks in the ROK.

## Author summary

The integration of genomic surveillance with spatial and temporal data offers a powerful approach to reconstruct viral transmission patterns across both geographic and temporal dimensions. Whole-genome sequencing is essential for elucidating the complex dynamics of viral evolution, genomic epidemiology, and the transmission pathways of emerging pathogens. Hantaan virus (HTNV) and the related genotypes classified under the species *Orthohantavirus hantaense,* primarily hosted by *Apodemus* species, are well-documented causative agents of hemorrhagic fever with renal syndrome (HFRS), posing a substantial global public health concern. Current evidence suggests that HTNV associated with *A. chejuensis* is the likely etiological agent responsible for HFRS cases on Jeju Island, Republic of Korea (ROK). This study conducted extensive HTNV surveillance, sampling 50 small mammals from five locations in Seogwipo-si and Jeju-si on Jeju Island between 2022 and 2023. Nine complete HTNV genomes were sequenced from lung tissues of *A. chejuensis*, constituting the first comprehensive genomic characterization of HTNV from Seogwipo-si (Hogeun-dong) and Jeju-si (Sangdae-ri). Phylodynamic analyses suggest evolutionary divergence and phylogeographic diversity, with four distinctive amino acid substitutions identified in HTNV genomes from Seogwipo-si. These findings contribute to our understanding of orthohantavirus genomic diversity and evolutionary dynamics, providing useful insights to inform public health strategies aimed at controlling HFRS outbreaks in the ROK.

## Introduction

Orthohantaviruses, members of the family Hantaviridae within the order Elliovirales, are enveloped RNA viruses with a negative-sense, single-stranded genome [1]. The genome is segmented into three parts: small (S), medium (M), and large (L), which encode a nucleocapsid protein (N), two membrane glycoproteins (Gn and Gc), and RNA-dependent RNA polymerase (RdRp), respectively. In Eurasia, orthohantaviruses are the primary cause of hemorrhagic fever with renal syndrome (HFRS), a disease with a fatality rate of 1–15% [2]. The viruses most commonly associated with HFRS include Hantaan virus (HTNV), Seoul virus (SEOV), Dobrava virus (DOBV), and Puumala virus, all of which belong to different *Orthohantavirus* species [3]. Humans are typically infected by inhalation of aerosolized viral particles from the saliva, urine, or feces of infected rodents [4].

Approximately 400 HFRS cases are reported annually in the Republic of Korea (ROK), with the case fatality rate averaging between 1% and 4% (accessible online at https://dportal.kdca.go.kr/pot/is/rginEDW.do). HTNV is the primary pathogen, accounting for 70% of these infections, followed by SEOV, which is responsible for 20%, with the remaining 10% attributed to unidentified viral agents [5]. HTNV was first isolated in 1978 from a striped field mouse (*Apodemus agrarius*) in the ROK, confirming its role as a causative agent of HFRS [6]. Currently, HTNV is classified into four distinct genotypes: two variants of the Hantaan virus associated with *A. agrarius* and *A. chejuensis* and the Soochong and Amur viruses, which are carried by *A. peninsulae* [6–10]. *A. agrarius*, the most prevalent rodent species, is widely distributed across the Korean Peninsula, whereas *A. chejuensis* is restricted to Jeju Island, the southernmost region of the ROK [11]. In 2012, Jeju virus was discovered and characterized from a shrew, *Crocidura shantungensis,* on Jeju Island [12,13]. However, its pathogenic potential remains uncertain. Based on current evidence, HTNV harbored by *A. chejuensis* is tentatively proposed as the etiological agent of HFRS on Jeju Island, ROK.

Next-generation sequencing (NGS) technologies have significantly advanced virology, enabling whole-genome sequencing, metagenomics, transmission tracking, genomic epidemiological studies, and vaccine development [14–18]. Sequencing the complete genomes of emerging viruses is crucial for clinical diagnostics, virulence assessment, and sources during outbreaks [19–21]. The MinION sequencer (Oxford Nanopore Technologies [ONT], London, UK) is a portable, long-read sequencing device that provides a compact, cost-effective alternative to conventional NGS platforms [22,23]. Nanopore sequencing has been successfully used to obtain full-length genomic sequences of two New World hantaviruses, Sin Nombre virus and Prospect Hill virus [24]. In addition, amplicon-based nanopore sequencing has been explored as a diagnostic method for rapidly generating near-complete genomic sequences of HTNV and SEOV from patients with HFRS and their rodent reservoirs [25–29].

Genomic surveillance, when combined with data on geographic location and sampling time, allows for the reconstruction of viral transmission patterns over space and time [30,31]. To achieve this, both heuristic and model-driven phylogeographic approaches have been developed, which offer a range of models to address key issues related to the spread and evolution of infectious diseases [32]. The coronavirus disease (COVID-19) pandemic has underscored the urgent need for rapid pathogen detection and monitoring through genomic surveillance integrated with epidemiological analysis [33]. During the 2014–2015 Ebola virus outbreak in West Africa, phylogenetic studies and evolutionary analyses were used in mapping viral transmission routes by integrating genomic data with spatial and temporal epidemiological information [34]. Similarly, the combination of clinical sequencing and epidemiology during the 2013–2015 Lassa virus outbreak provided real-time insights into transmission patterns, particularly rodent-to-human spread [35]. These approaches, which utilize both discrete and continuous geographic data, are becoming increasingly important in viral phylodynamics, offering a deeper understanding of the evolution and spread [36,37].

In this study, an epidemiological survey was conducted to assess the serological and viral prevalence of hantaviruses in rodents and shrews captured on Jeju Island, ROK, between 2022 and 2023. Amplicon-based NGS was employed to sequence the complete genome of HTNV to better understand its evolutionary diversification in Seogwipo-si and Jeju-si, Jeju Island. Phylodynamic analyses were performed to examine the evolutionary divergence and geographic variation of HTNV across Jeju Island, with an emphasis on identifying the key mutation dynamics. This report provides important insights into the genomic surveillance, phylogenetic diversity, and evolutionary dynamics of orthohantaviruses that are essential for developing effective strategies to mitigate HFRS outbreaks in the ROK.

## Methods

### Ethics statement

All small mammals were handled in accordance with the ethical guidelines approved by the Korea University Institutional Animal Care and Use Committee (Approval number: KUIACUC #2022–0034). All necropsies and laboratory experiments were performed in a biosafety level 3 facility at the Korea University College of Medicine, Seoul, ROK.

### Sample collection

Between 2022 and 2023, small mammals—including rodents and shrews—were captured at five locations on Jeju Island, ROK: Hogeun-dong and Seohong-dong in Seogwipo-si, and Bongseong-ri, Sangdae-ri, and Ora-dong in Jeju-si. The procedures for trapping and transportation have been described previously [38]. Briefly, field sampling was conducted once annually at each site over the study period, with each trapping session lasting exactly two consecutive nights. At each location, 20–25 collapsible, live-capture Sherman traps (7.7 × 9 × 23 cm; H.B. Sherman, Tallahassee, FL, USA) were deployed each afternoon along transects spaced at 4–5 meter intervals and retrieved the following morning. Traps were placed in ecotones and transitional zones across a variety of habitats, including unmanaged grasslands, herbaceous vegetation, farmlands, and forest edges. Given the five sites sampled over two years, with two-night trapping sessions at each visit, the total trapping effort was estimated at approximately 400–500 trap-nights, depending on the number of traps deployed per site. Captured animals were individually labeled, placed in secure transport containers, and transferred to Korea University in Seoul. Upon arrival, individuals were humanely euthanized and identified to species based on external morphological characteristics; sex and body weight were also recorded. Whole blood and internal organs (lung, liver, spleen, and kidney) were aseptically collected in accordance with protocols approved by the KUIACUC and stored at −80°C until further analysis.

### Mitochondrial DNA (mtDNA) analysis

Total DNA was extracted from the homogenized liver tissues using either the High Pure PCR Template Preparation Kit (Roche, Basel, Switzerland) or TRI Reagent Solution (Ambion, Austin, Texas, USA) according to the manufacturer’s instructions. To verify the taxonomic classification of small mammals, PCR amplification was performed targeting the mtDNA cytochrome *b* (CYTB) gene. The PCR employed universal primers for mammals and followed the cycling conditions outlined previously [39].

### Indirect immunofluorescence antibody test

Serum and heart fluids were diluted with phosphate buffered saline (PBS) to final dilution factors of 1:32 and 1:2, respectively. The diluted samples were then applied to ten-well slides (PTFE Printed Slide 10 well; Electron Microscopy Science, USA) containing acetone-fixed HTNV-infected Vero E6 cells and incubated at 37°C for 30 min. Following incubation, the plates were washed sequentially with PBS and distilled water (D.W.). The cells were then treated with fluorescein isothiocyanate (FITC)-conjugated goat antibodies specific to mouse immunoglobulin G (IgG) for rodents or mouse or rat IgG for shrews (MP Bio, CA, USA). The slides were then incubated at 37°C for 30 min and washed. Hantavirus-specific fluorescence was identified using a fluorescence microscope (Axio Scope; Zeiss, Berlin, Germany). Antibody titers were quantified as the highest dilution of the sample that exhibited weak yet specific fluorescence, as determined through a two-fold serial dilution.

### Reverse transcription-polymerase chain reaction (RT-PCR)

Total RNA was isolated from 50–100 mg of homogenized rodent lung tissue using TRI Reagent Solution (Ambion), in accordance with the manufacturer’s protocol. cDNA was synthesized from 1 µg of total RNA employing the High-Capacity RNA-to-cDNA Kit (Applied Biosystems, Foster City, CA, USA), using a random hexamer primer in conjunction with OSM55 (5′–TAG TAG TAG ACT CC–3′). Subsequent amplification of the target regions was conducted through first-round and nested PCR in a total volume of 25 µL. Each reaction mixture comprised 0.625 U of Ex Taq DNA polymerase (TaKaRa Bio, Japan), 2.5 µL of 10 × reaction buffer, 2 µL of a 2 mM dNTP mix, 10 pmol of each primer (final concentration: 0.4 µM), and 1.5 µL of the cDNA template. Thermal cycling conditions included an initial denaturation at 94°C for 5 min, followed by six cycles of denaturation at 94°C for 30 s, annealing at 37°C for 40 s, and extension at 72°C for 1 min. This was succeeded by 32 cycles of denaturation at 94°C for 30 s, annealing at 42°C for 40 s, and extension at 72°C for 1 min, concluding with a final extension step at 72°C for 5 min. PCR amplifications were carried out using the ProFlex PCR System (Life Technologies, CA, USA). Amplified products were purified with the MinElute PCR Purification Kit (Qiagen, Hilden, Germany). Bidirectional Sanger sequencing was performed using the BigDye Terminator v3.1 Cycle Sequencing Kit (Applied Biosystems) on an ABI 3730XL DNA Analyzer (Applied Biosystems). The primers used for amplification have been previously described [9].

### Quantitative PCR (qPCR)

qPCR was conducted in a 10 µL reaction mixture consisting of 5 µL of SYBR Green PCR Master Mix (Applied Biosystems), 3 µL of D.W., 1 µL of cDNA, and 5 pmol of each primer (final concentration: 0.4 µM), using the QuantStudio 5 Flex Real-Time PCR System (Applied Biosystems). The cycling conditions and primer sequences have been previously described [9]. Viral copy numbers were quantified by generating a standard curve from recombinant plasmid DNA containing the S segment of HTNV, as outlined in prior studies [40].

### Multiplex PCR-based nanopore sequencing

cDNA was amplified using previously described multiplex HTNV-specific primer mixtures designed for southern Korean strains, in combination with the Solg 2X Uh-Taq PCR Smart Mix (Solgent, Daejeon, ROK), following the protocol established by Prayitno et al (2024) [27]. Library preparation was performed using a Native Barcoding Kit 24 V14 (SQK-NBD 114.24; ONT). Following end preparation, the libraries were purified using AMPure XP beads (Beckman Coulter, CA, USA). The purified barcoded libraries were pooled, ligated to sequencing adapters, and subsequently sequenced on a MinION MK1C device (ONT) using a FLO-MIN114 (R10.4.1) flow cell for 14 h.

Raw reads were demultiplexed and adapter-trimmed using Porechop (v0.9.0), and only reads with a minimum Q-score of 7 were retained. Index sequences were removed, and to minimize the risk of artificial primer-derived sequence similarity, the first and last 20 nucleotides at the 5′ and 3′ ends of each read were trimmed using CLC Genomics Workbench (v22.0.2; Qiagen, Hilden, Germany). Filtered reads were then mapped to the HTNV reference genome (strain Ac20–5) using the following alignment parameters: match score = 2, mismatch cost = 3, length fraction = 0.7, similarity fraction = 0.8, and non-specific match handling = ignore. Genome coverage depth was subsequently calculated, and consensus sequences were generated based on majority-rule nucleotide calling at positions with a minimum depth of 5 × ; positions with coverage <5 × were excluded and denoted as ‘N’. Indels and frameshift errors within open reading frames were manually corrected according to previously established error correction criteria by comparing the mapped reads with the reference genome [25]. Genome regions not covered by the reads were amplified and confirmed using conventional nested RT-PCR. Final consensus sequences were deposited in GenBank.

### Rapid amplification of cDNA ends (RACE) PCR

To acquire the 3’ and 5’ terminal sequences of the HTNV genome, RACE PCR was carried out using the 3’- and 5’-RACE System for Rapid Amplification of cDNA Ends, Version 2.0 (Invitrogen, Carlsbad, CA, USA), following the manufacturer’s protocol.

### Phylogenetic analysis

The genomic sequences of HTNV tripartite segments were aligned using the ClustalW algorithm in MegAlign (v5; DNASTAR, Madison, WI, USA). The most appropriate substitution model was determined to be GTR + G + I using the MEGA 7 software. Phylogenetic trees for each segment were constructed using Bayesian Evolutionary Analysis by Sampling Trees (BEAST v2.6.7) under a strict molecular clock model [41]. The analysis was conducted over 80 million iterations, ensuring an effective sample size of greater than 200.

### Network analysis

Phylogenetic networks were constructed using the median-joining algorithm implemented in Network software (version 10.2.0.0; Fluxus Engineering, Cambridge, UK), with the epsilon set to 0 to reduce reticulations and simplify network topology. The networks were generated from multiple sequence alignments of HTNV genomes, including 55 sequences for the S and M segments and 51 sequences for the L segment. Analyses were based on aligned nucleotide positions spanning the S segment (49–1,343 nt), the M segment (1–3,431 nt), and the L segment (28–6,506 nt). The median-joining method integrates minimum spanning trees with inferred median vectors to represent potential ancestral relationships and mutational steps among sequences. Lineages were delineated based on genetic distance, resulting in the identification of two major groups. Network visualization was performed using Network Publisher (version 2.1.2.5) and finalized in Adobe Illustrator 2021.

### Amino acid substitution analysis

The amino acid sequences of the three HTNV genes were aligned using the ClustalW method in MegAlign (v5; DNASTAR). Amino acid mutations in HTNV were identified for each variant by comparison with the reference HTNV Ac20–5. The lollipop plot was visualized using the *dplyr* and *ggplot2* packages in R version 4.3.3.

## Results

### Field trapping for small mammals on Jeju Island, 2022–2023

**Fig 1:**
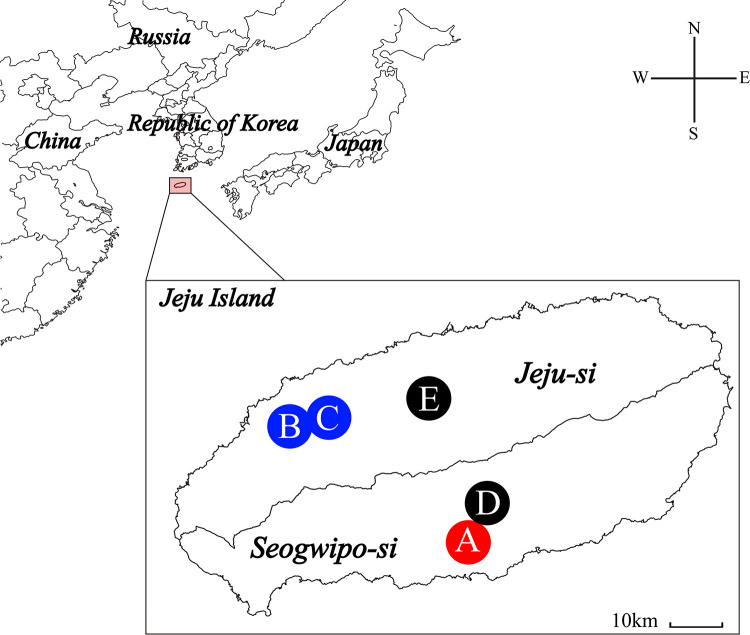
Geographic locations of Hantaan virus (HTNV) carried by *Apodemus chejuensis* captured on Jeju Island, Republic of Korea, in 2022–2023. The map delineates the five sampling sites: **(A)** Hogeun-dong, **(B)** Bongseong-ri, **(C)** Sangdae-ri, **(D)** Seohong-dong, and **(E)** Ora-dong. Locations where HTNV was detected are marked by red and blue circles, whereas black circles denote sites where HTNV was not detected. The initial map was generated using Quantum Geographical Information System (QGIS) 3.10 for Windows and subsequently refined in Adobe Illustrator 2020 (Adobe Systems Incorporated, California, USA). The base layer of the map was sourced from https://www.naturalearthdata.com/ and is freely available for use in any project without the need for permission.

We collected 50 small mammals from five locations: across Seogwipo-si (Hogeun-dong and Seohong-dong) and Jeju-si (Bongseong-ri, Sangdae-ri, and Ora-dong), Jeju Island, ROK, during 2022–2023 (Fig 1). The captured animals included 47 *Apodemus chejuensis* (94%) and three *Crocidura shantungensis* individuals (6%) (S1 Table). The species identification of *A. chejuensis* was validated by phylogenetic analysis based on mitochondrial sequencing (S1 Fig).

### Epidemiological surveillance of HTNV

Serological testing of *A. chejuensis* revealed a positivity rate of 34% (16/47), whereas HTNV RNA was detected in 27.7% (13/47) (Table 1). HTNV RNA positivity rates were 27.6% (8/29) in Hogeun-dong, Seogwipo-si; 57.1% (4/7) in Bongseong-ri, Jeju-si; and 12.5% (1/8) in Sangdae-ri, Jeju-si. Males exhibited a higher viral positivity rate (37.5%, 9/24) than females (17.4%, 4/23). The prevalence also increased with weight: 17.4% (4/23) for 21–30 g, 54.5% (6/11) for 31–40 g, and 75% (3/4) for >40 g. No HTNV RNA was detected in rodents weighing 11–20 g. The epidemiological characteristics of HTNV-infected *A. chejuensis* are shown in S2 Table. In this study, the shrews tested negative in both serological and molecular assays.

**Table 1:** Serological and molecular prevalence of Hantaan virus (HTNV) in *Apodemus chejuensis* collected on Jeju Island, Republic of Korea, 2022– 2023.

Characteristic		Number of captured rodents	HTNV Seroprevalence (%)	HTNV Prevalence (%)^a^
**Region, n = 47**				
Seogwipo-si	Hogeun-dong	29	9/29 (31)	8/29 (27.6)
	Seohong-dong	1	0/1	0/1
Jeju-si	Bongseong-ri	7	6/7 (85.7)	4/7 (57.1)
	Ora-dong	2	0/2	0/2
	Sangdae-ri	8	1/8 (12.5)	1/8 (12.5)
**Sex, n = 47**				
Male		24	11/24 (45.8)	9/24 (37.5)
Female		23	5/23 (21.7)	4/23 (17.4)
**Weight, g, n = 47**				
11-20		9	2/9 (22.2)	0/9
21-30		23	5/23 (21.7)	4/23 (17.4)
31-40		11	6/11 (54.5)	6/11 (54.5)
> 40		4	3/4 (75)	3/4 (75)
**Total**		**47**	**16/47 (34)**	**13/47 (27.7)**

^a^HTNV prevalence was confirmed using a nested reverse transcription-polymerase chain reaction.

### Determination of HTNV RNA copy numbers from *A. chejuensis* lung tissues

HTNV RNA loads in the lung tissues of 13 *A. chejuensis* were measured (S3 Table). Five rodents (Ac23–18, Ac23–20, Ac23–15, Ac23–19, and Ac22–24) exhibited the highest viral RNA loads, with the Ct values ranging from 15.9 to 20.3, corresponding to 10⁵–10⁶ copies/μL. Three individuals (Ac23–1, Ac23–17, and Ac23–19) showed Ct values ranging from 23.0 to 24.2, indicating viral RNA amounts of 10⁴–10⁵ copies/μL. Ac23–12 and Ac23–22 had viral RNA quantities of 10²–10³ copies/μL, with Ct values between 27.2 and 29.0. Additionally, three *A. chejuensis* strains (Ac23–14, Ac22–20, and Ac22–23) exhibited the lowest viral loads, ranging from 0 to 1 copy, with Ct values >38.0.

### Whole-genome sequencing of HTNV

Using amplicon-based nanopore sequencing, nearly complete genome sequences of HTNV were obtained from lung tissues of *A. chejuensis* collected from Jeju Island, ROK, between 2022 and 2023 (Table 2). Five rodents (Ac23–18, Ac23–20, Ac23–15, Ac23–19, and Ac22–24) exhibited the highest viral loads, containing 10⁵ to 10⁶ copies/µL, with genome coverages of 96.4%–98.5%, 97.5%–99.3%, and 97.1%–99.6% for the S, M, and L segments, respectively. Three individuals (Ac23–1, Ac23–17, and Ac23–19) with RNA copy numbers ranging from 10⁴ to 10⁵ copies/µL showed coverages of 98.5%, 95.3%–99.8%, and 95.8%–98.6% for the S, M, and L segments, respectively. Two variants (Ac23–12 and Ac23–22) with 10² to 10³ copies/µL displayed coverages of 95.7%–96.3%, 87.6%–91.6%, and 93.7%–93.8% for the S, M, and L segments, respectively. The three samples with the lowest viral RNA amounts (0–1 copy/µL; Ac23–14, Ac22–20, and Ac22–23) exhibited genome coverages of 86.0%–96.3%, 80.8%–91.2%, and 71.8%–93.0% for the S, M, and L segments, respectively. The average number of mapped viral reads and sequencing depths are listed in S4 Table.

**Table 2:** Genome coverage rate of Hantaan virus (HTNV) in lung tissues of *Apodemus chejuensis* collected from Jeju Island, based on viral RNA copy number.

Viral RNA copy number (copies/uL)	Sample	Collection site		HTNV genomes, coverage rate^a^		
				S segment	M segment	L segment
10^5^ to 10^6^	Ac23–18	Seogwipo-si	Hogeun-dong	96.4	97.5	97.1
	Ac23–20	Seogwipo-si	Hogeun-dong	98.1	99.1	99.6
	Ac23–15	Seogwipo-si	Hogeun-dong	98.5	99.1	98.8
	Ac23–19	Seogwipo-si	Hogeun-dong	98.5	99.1	98.7
	Ac22–24	Jeju-si	Bongseong-ri	98.5	99.3	99.6
10^4^ to 10^5^	Ac23–1	Jeju-si	Sangdae-ri	98.5	96.5	98.6
	Ac23–17	Seogwipo-si	Hogeun-dong	98.5	99.8	95.8
	Ac22–19	Jeju-si	Bongseong-ri	98.5	95.3	98.3
10^2^ to 10^3^	Ac23–12	Seogwipo-si	Hogeun-dong	96.3	87.6	93.7
	Ac23–22	Seogwipo-si	Hogeun-dong	95.7	91.6	93.8
0 to 1	Ac23–14	Seogwipo-si	Hogeun-dong	96.3	91.2	93.0
	Ac22–20	Jeju-si	Bongseong-ri	91.9	80.8	73.9
	Ac22–23	Jeju-si	Bongseong-ri	86.0	81.3	71.8

^a^Coverage was estimated by mapping viral reads to the HTNV reference genome (strain Ac20–5) using CLC Genomics Workbench (v22.0.2; Qiagen). Nucleotide positions with coverage depth <5 were excluded and denoted as ‘N’. The coverage rate for each segment was calculated using the formula: (covered genome length/ reference genome size) × 100.

Ac, *Apodemus chejuensis*.

Nine full-length genomic sequences of HTNV were recovered from rodents captured at three locations: Hogeun-dong in Seogwipo-si and Sangdae-ri and Bongseong-ri in Jeju-si, Jeju Island, ROK, in 2022–2023. Gaps in the HTNV tripartite RNA genome sequences were filled using conventional nested RT-PCR. To achieve a whole-genome sequence of HTNV, 5′ and 3′ RACE PCR was conducted on HTNV Ac23–18, a specimen from Seogwipo-si on Jeju Island with the highest viral copy number. The 5′ terminal sequence of HTNV tripartite RNA was determined to be 5′-GGA GUC UAC UAC UA-3′, whereas the 3′ end sequence was identified as 5′-UAG UAG UAU GCU CC-3, ’ corresponding to conserved termini sequences of the reference genome sequence (HTNV Ac20–5) [9]. Terminal sequences of the remaining HTNV specimens were recovered empirically.

### Genome comparison of HTNV

Nucleotide comparison analysis revealed that the HTNV genome shared 99.2% identity in the S segment, 99.1%–99.2% identity in the M segment, and 99.4–99.8% identity in the L segment with the reference HTNV Ac20–5, which was originally detected in *A. chejuensis* captured in Ora-dong, Jeju-si, ROK (S5 Table). The amino acid sequences showed similarities of 99.4%–99.6% for the N protein, 99.5%–99.6% for the glycoproteins, and 99.5%–99.6% for the RdRp.

### Phylodynamic analysis of HTNV

**Fig 2:**
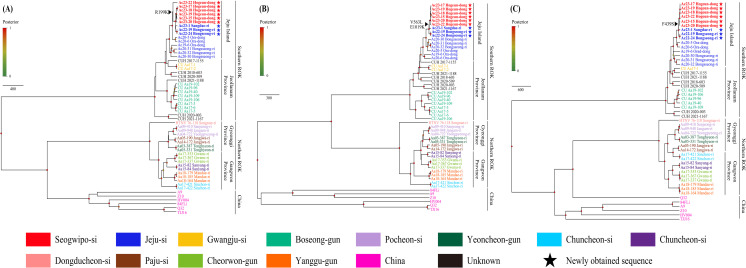
Phylodynamic analysis of Hantaan virus (HTNV) S, M, and L segments from *Apodemus chejuensis* captured on Jeju Island, Republic of Korea (ROK), in 2022–2023. Nine whole-genome sequences of HTNV were newly obtained in this study. Phylogenetic inference was performed using an alignment based on the HTNV **(A**) S (49–1,343 nt), **(B)** M (1-3,431 nt), and **(C)** L (28-6,506 nt) genomic sequence, using the strict clock method within the Bayesian Evolutionary Analysis Sampling Trees (BEAST, v2.6.7). A total of 80 million iterations were conducted to ensure an effective sample size greater than 200. Posterior probabilities are displayed at the tips of each node, with colored circles indicating nodes with posterior values. The arrowhead marks the branch point of differentiation associated with a key amino acid variation in each segment, which was notably found in Seogwipo-si, Jeju Island, ROK. Sequence colors correspond to their respective collection sites at the city level, with the newly acquired HTNV sequences from this study denoted by stars. Accession numbers for the hantaviral sequences from this study are listed in S6 Table.

A phylodynamic analysis was conducted to explore the genetic diversity and mutation dynamics among the tripartite genomes of HTNV derived from *A. chejuensis* collected from Jeju Island, ROK, from 2022 to 2023 (Fig 2). Phylogenetic analysis of nine HTNV variants, including Ac23–15, Ac23–17, Ac23–18, Ac23–19, Ac23–20, and Ac23–22 from Hogeun-dong in Seogwipo-si; Ac23–1 from Sangdae-ri; and Ac22–19 and Ac22–24 from Bongseong-ri in Jeju-si, demonstrated consistent clustering into a homologous genetic group along with other HTNV variants collected from Jeju Island, ROK. However, discordance in evolutionary divergence was observed within the S segment of the HTNV variant Ac23–22 from Hogeun-dong in Seogwipo-si compared to the M and L segments.

Moreover, four unique amino acid substitutions were identified in the tripartite genomes of HTNV variants from Seogwipo-si (Table 3 and S2 Fig). These mutations comprised one in the S segment (N protein; R199K), two in the M segment (glycoproteins; V563L and E1019K), and one in the L segment (RdRp; F439S) when compared to the corresponding sequences of HTNV variants from Jeju-si.

**Table 3:** Key amino acid substitutions in the S, M, and L segments of Hantaan virus (HTNV) carried by *Apodemus chejuensis* collected on Jeju Island, Republic of Korea.

Segment	Amino acid substitution^a^			Variation site
	Seogwipo-si	Jeju-si	Reference	
S	K	R	R	199
M	L	V	V	563
	K	E	E	1019
L	S	F	F	439

^a^, Key amino acid substitutions were determined in the genomic sequences of HTNV from Seogwipo-si and compared to the reference HTNV Ac20–5.

### Phylogeographic network analysis of HTNV

**Fig 3:**
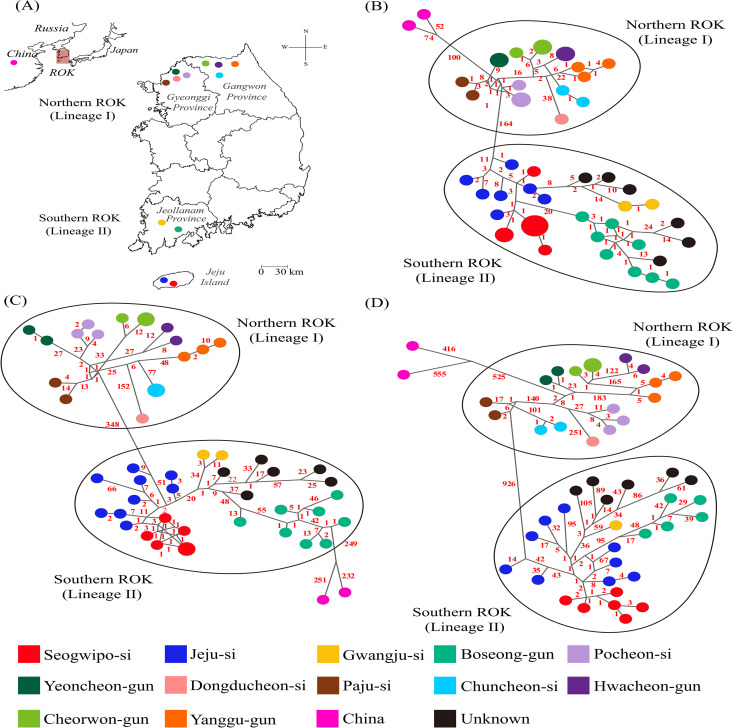
Phylogeographic network analysis of Hantaan virus (HTNV) from *Apodemus chejuensis* collected on Jeju Island, Republic of Korea (ROK), in 2022–2023. The phylogeographical network was generated from alignments of HTNV tripartite genomes for 55 genomic sequences (for S and M segments) and 51 genomic sequences (for L segment). **(A)** The geographic distributions of HTNV variants are indicated by colored circles at the collection sites on the map. The initial map was generated using Quantum Geographical Information System (QGIS) 3.10 for Windows. The base layer of the map was sourced from https://www.naturalearthdata.com/ and is freely available for use in any project without the need for permission. Networks were constructed using the median-joining method in Network software (version 10.2.0.0; Fluxus Engineering, Cambridge, UK) with an epsilon value of 0, based on the HTNV **(B)** S (49–1,343 nt), **(C)** M (1–3,431 nt), and **(D)** L (28–6,506 nt) segments. Color-coded symbols represent individual HTNV variants according to their collection sites, with circle sizes proportional to the connection frequency between viruses. The nodes illustrate the relationships among viral strains, with red numbers on the nodes indicating the total nucleotide mutations between strains. Lineage 1 spans regions in northern ROK, including Pocheon-si, Yeoncheon-gun, Dongducheon-si, and Paju-si in Gyeonggi Province, and Chuncheon-si, Hwacheon-gun, Cheorwon-gun, and Yanggu-gun in Gangwon Province. Lineage 2 covers areas in southern ROK, including Seogwipo-si and Jeju-si on Jeju Island, as well as Gwangju-si and Boseong-gun in Jeollanam Province. HTNV genomes from China were used as the outgroup in this study. Accession numbers for the hantaviral sequences from this study are listed in S6 Table.

Network analyses revealed a well-supported evolutionary divergence and genetic diversity among the HTNV S, M, and L genomes across various geographic regions of Jeju Island and the Korean Peninsula (Fig 3). Two distinct phylogenetic lineages were identified in the ROK. Lineage 1 encompasses regions in the northern ROK, including Pocheon-si, Yeoncheon-gun, Dongducheon-si, and Paju-si in Gyeonggi Province, and Chuncheon-si, Hwacheon-gun, Cheorwon-gun, and Yanggu-gun in Gangwon Province. Conversely, lineage 2 was associated with the southern regions of the ROK, including Seogwipo-si and Jeju-si on Jeju Island, and Gwangju-si and Boseong-gun in Jeollanam Province. These two lineages were defined based on consistent phylogeographic clustering and regional separation observed across all three genomic segments. The mean nucleotide divergence between Lineage 1 and Lineage 2 ranged from approximately 12% to 18%, depending on the segment.

## Discussion

Genomic surveillance, in conjunction with epidemiology, has played a crucial role in informing public health strategies for HFRS by elucidating the transmission pathways between patients and their infection sources [42]. For instance, in 2005, clinical sequencing of HTNV from four U.S. army soldiers diagnosed with HFRS during their deployment with U.S. Forces Korea (USFK) demonstrated a phylogeographic association between the viral sequences and those recovered from rodents captured at military training sites where the soldiers had been active [43]. Subsequent genomic investigations conducted between 2013 and 2015, where near-complete HTNV genome sequences from both ROK and USFK soldiers, further substantiated these epidemiological correlations [44]. Notably, active surveillance efforts involving targeted rodent trapping at sites suspected of orthohantavirus outbreaks linked to HFRS have allowed researchers to distinguish between viral lineages within short distances (approximately 5 km), making it possible to track specific strains of the virus and identify likely sites of exposure more accurately [45]. In the present study, nine whole-genome sequences of HTNV were obtained from *A. chejuensis* collected from Seogwipo-si and Jeju-si, Jeju Island, ROK, between 2022 and 2023. These findings represent the first comprehensive characterization of the complete genome sequences of HTNV from Seogwipo-si (Hogeun-dong) and Jeju-si (Sangdae-ri), Jeju Island. Jeju Island has been recognized as a HFRS-endemic region since the official identification of the first clinical case in 2005 (accessible online at https://dportal.kdca.go.kr/pot/is/rginEDW.do). To date, 28 clinical cases have been reported on this island. However, only a single clinical case has been documented as a case report on the island, without any molecular clues provided by genomic epidemiology owing to the lack of direct clinical sequencing of the patient samples [46]. Recently, genomic surveillance revealed the etiological agent and clinical characteristics of HFRS in the southern areas of the ROK (Gwangju Metropolitan City and Boseong-gun), providing molecular evidence of a novel HTNV genotype that causes hantaviral diseases, which was clustered as lineage 2 in our study [27]. To clarify the transmission dynamics and pathogenicity of HTNV, further studies should focus on sequencing and characterization of the clinical features of HFRS in patients from Jeju Island, ROK.

In this study, we conducted a comparative genomic analysis to explore the evolutionary dynamics of HTNV carried by *A. chejuensis* on Jeju Island, ROK. Phylogeographic analyses revealed geographically structured evolutionary divergence among HTNV variants sampled from different regions of the island. Phylogenetic incongruence was observed in the tripartite genome of HTNV Ac23–22, with the S segment diverging from the cluster formed by its M and L segments, which grouped with strains from Hogeun-dong, Seogwipo-si. Such patterns may result from segment-specific evolutionary pressures or past reassortment events, as genome reassortment among closely related strains has been reported in hantaviruses [47–49]. In the ROK, a putative hybrid zone has been proposed where lineage overlap may facilitate segment exchange [50,51]. GiRaF analysis did not detect any statistically supported reassortment events in our dataset. While earlier studies have provided an important genomic foundation, the number of complete HTNV sequences from Jeju Island remains limited. Continued efforts to expand genomic sampling will be essential for refining our understanding of HTNV phylogeography and evolutionary dynamics in this under-sampled region.

The classification of DOBV lineages (Dobrava, Sochi, Kurkino, and Saaremaa) is primarily based on distinctions in their phylogenetic relationships, associated rodent reservoirs, and geographic distribution [52]. Despite the high degree of genetic similarity among these genotypes, they elicit HFRS of varying severities. The case fatality rate (CFR) of infections attributed to the Dobrava genotype, which is carried by *A. flavicollis*, ranges between 10% and 12% [53]. Conversely, the HFRS cases linked to the Sochi genotype hosted by *A. ponticus* exhibited a CFR of approximately 6% [54]. Moreover, orthohantavirus outbreaks related to the Kurkino genotype harbored by *A. agrarius* demonstrated a CFR of less than 1% in regions of European Russia. Notably, infections caused by the Saaremaa genotype, which is also carried by *A. agrarius*, tend to be subclinical [55]. Although these DOBV lineages differ in clinical severity and host specificity, genetic diversity also plays a key role in shaping the pathogenic potential of other orthohantaviruses such as HTNV. In this study, pairwise sequence comparisons revealed high nucleotide and amino acid identity between the newly obtained HTNV genomes from Seogwipo-si and the reference strain Ac20–5, with divergence not exceeding 1% across all segments. However, four unique amino acid substitutions were identified in the HTNV genomes from Seogwipo-si—R199K in the S segment (N protein), V563L and E1019K in the M segment (glycoproteins), and F439S in the L segment (RdRp)—compared to those from Jeju-si. These mutations, while possibly resulting from natural genetic variability, were geographically distinct and may warrant further investigation. Although HTNV strains from Jeju Island have not yet been isolated, future studies incorporating virus isolation and functional analyses will be important to determine the potential phenotypic relevance of these substitutions.

While this study provides valuable genomic insights into HTNV diversity on Jeju Island, several limitations should be acknowledged. 1) The total number of small mammals collected was limited, despite a systematic trapping design across ecologically distinct sites. As a result, the current findings offer only a preliminary indication of HTNV prevalence, and more extensive, longitudinal sampling will be necessary to better define its geographic distribution and infection dynamics. 2) Several amino acid substitutions were identified, but their potential structural or functional implications could not be evaluated within the scope of this study. Future work should incorporate protein modelling and virus isolation followed by experimental validation to clarify the biological significance of these variations. 3) The phylodynamic analysis was based on a relatively small number of sequences from a narrow temporal and geographic window, limiting the resolution of inferred evolutionary patterns. Broader and longer-term sampling will be essential to strengthen future phylodynamic inferences. 4) The origin of HTNV strains detected on Jeju Island remains unclear. While sequence similarity with strains from the Korean mainland suggests a possible introduction from the peninsula, phylogeographic interpretation is limited by the lack of comparative genomic data from nearby regions, particularly southeastern China. Further regional sampling will be needed to better clarify the virus’s evolutionary history.

In conclusion, nine complete HTNV genomic sequences were acquired from rodents collected from Jeju Island, ROK, between 2022 and 2023. This is the first complete characterization of HTNV genomes from Seogwipo-si (Hogeun-dong) and Jeju-si (Sangdae-ri), enhancing the phylogeographic resolution of the region. Phylodynamic analysis revealed a well-supported evolutionary divergence and geographic diversity across Jeju Island, with four unique amino acid substitutions identified in the HTNV genomes from Seogwipo-si. These findings provide valuable insights into the genomic surveillance, phylogenetic diversity, and evolutionary dynamics of orthohantaviruses, which are crucial for developing effective strategies to mitigate HFRS outbreaks in the ROK.

## Supporting information

S1 Fig Phylogeny based on the mitochondrial DNA cytochrome *b* (CYTB) gene of *Apodemus chejuensis* collected on Jeju Island, Republic of Korea (ROK), in 2022–2023. A phylogenetic tree generated from the mitochondrial DNA CYTB gene sequences (686 bps) of *A. chejuensis* captured on Jeju Island, ROK. A phylogenetic analysis was conducted with the HKY + G substitution model using the maximum likelihood method in MEGA 7.0. The branch lengths in the phylogenetic tree correspond to the number of nucleotide substitutions, while the vertical distances are adjusted for improved visual clarity. Bootstrap probabilities, calculated from 1,000 iterations, are indicated at each node. In this figure, the genomic sequences of the CYTB gene from *A. chejuensis* are displayed, with the newly obtained sequences highlighted in bold red font.
(TIF)

S2 Fig Amino acid substitutions in the tripartite genomes of Hantaan virus (HTNV) carried by *Apodemus chejuensis* collected on Jeju Island, Republic of Korea, in 2022–2023. The lollipop plot illustrates the positions of amino acid mutations in the nine HTNV sequences obtained in this study, compared to the reference HTNV Ac20–5 genomic sequences. The plot was visualized using the *dplyr* and *ggplot2* packages in R version 4.3.3. Each node represents a point of amino acid variations, with the node length indicating the frequency of HTNV amino acid substitutions across different rodent collection sites. The color-coded bars correspond to the tripartite segments: S segment (blue), M segment (green), and L segment (red). Red nodes highlight key amino acid changes uniquely found in sequences from Seogwipo-si. The scale bar represents the length of HTNV amino acids and nucleotides.
(TIF)

S1 Table Species composition of 50 small mammals captured on Jeju Island, Republic of Korea, in 2022–2023. (PDF)

S2 Table Characteristics of Hantaan virus (HTNV)-infected *Apodemus chejuensis* collected on Jeju Island, Republic of Korea, in 2022–2023. (PDF)

S3 Table Hantaan virus RNA copy numbers from the lung tissues of *Apodemus chejuensis* collected on Jeju Island, Republic of Korea. (PDF)

S4 Table Summary of mapped reads and sequencing depth of amplicon-based nanopore sequencing for Hantaan virus harbored by *Apodemus chejuensis* collected on Jeju Island, Republic of Korea, in 2022–2023. (PDF)

S5 Table Nucleotide and amino acid sequence similarities of Hantaan virus (HTNV) carried by *Apodemus chejuensis* collected on Jeju Island, Republic of Korea, in 2022–2023. (PDF)

S6 Table Accession numbers of genomic sequences of Hantaan virus (HTNV) S, M, and L segments in the current study. (PDF)
