# Supplementary figures and images for: Phylogenetic diversity and molecular evolution of Hantaan virus harbored by Apodemus chejuensis on Jeju Island, Republic of Korea, 2022–2023
Source: PLoS Negl Trop Dis. 2025 Aug 19;19(8):e0013459. doi: 10.1371/journal.pntd.0013459 (PMC12373272; doi:10.1371/journal.pntd.0013459)

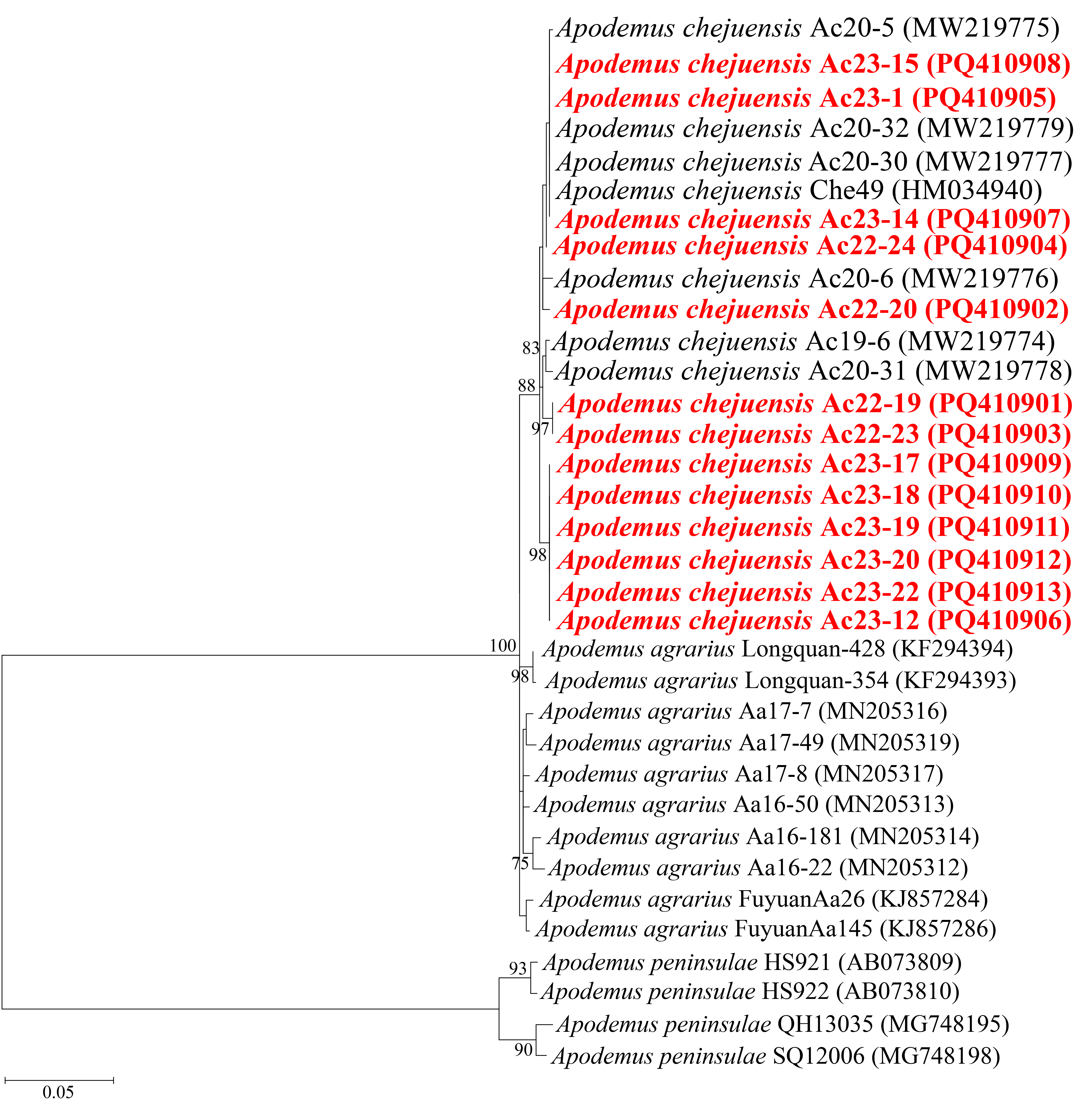

Supplement: S1 Fig — A phylogenetic tree generated from the mitochondrial DNA CYTB gene sequences (686 bps) of A. chejuensis captured on Jeju Island, ROK. A phylogenetic analysis was conducted with the HKY + G substitution model using the maximum likelihood method in MEGA 7.0. The branch lengths in the phylogenetic tree correspond to the number of nucleotide substitutions, while the vertical distances are adjusted for improved visual clarity. Bootstrap probabilities, calculated from 1,000 iterations, are indicated at each node. In this figure, the genomic sequences of the CYTB gene from A. chejuensis are displayed, with the newly obtained sequences highlighted in bold red font. (TIF) [file pntd.0013459.s001.tif]
