# Supplementary material for: Phylogenetic diversity and molecular evolution of Hantaan virus harbored by Apodemus chejuensis on Jeju Island, Republic of Korea, 2022–2023
Source: PLoS Negl Trop Dis. 2025 Aug 19;19(8):e0013459. doi: 10.1371/journal.pntd.0013459 (PMC12373272; doi:10.1371/journal.pntd.0013459)
Supplement: S1 Table — (PDF) [file pntd.0013459.s003.pdf]

S1 Table. Species composition of 50 small mammals captured on Jeju Island, Republic of Korea, in 2022–2023.

Species	Trapping location (GPS coordinates)					Total (%)
	Jeju-si			Seogwipo-si		
	Bongseong-ri (33°24'53"N 126°18'11"E)	Ora-dong (33°27'13"N 126°30'38"E)	Sangdae-ri (33°24'52"N 126°18'8"E)	Hogeun-dong (33°15'3"N 126°32'38"E)	Seohong-dong (33°15'13"N 126°32'46"E)	
Apodemus chejuensis	7	2	8	29	1	**47 (94)**
Crocidura shantungensis	-[a]	-[a]	1	2	-[a]	**3 (6)**
**Total**	**7**	**2**	**9**	**31**	**1**	**50 (100)**

[a]: not collected. GPS, global positioning system.
