# Supplementary material for: Phylogenetic diversity and molecular evolution of Hantaan virus harbored by Apodemus chejuensis on Jeju Island, Republic of Korea, 2022–2023
Source: PLoS Negl Trop Dis. 2025 Aug 19;19(8):e0013459. doi: 10.1371/journal.pntd.0013459 (PMC12373272; doi:10.1371/journal.pntd.0013459)
Supplement: S2 Table — (PDF) [file pntd.0013459.s004.pdf]

S2 Table. Characteristics of Hantaan virus (HTNV)-infected *Apodemus chejuensis* collected on Jeju Island, Republic of Korea, in 2022–2023.

Sample	Collection date	Collection site	Sex	Weight (g)	Anti-HTNV IgG titer	RT-PCR
Ac23-18	Apr. 19, 2023	Hogeun-dong, Seogwipo-si	M	39.5	512[a]	Pos
Ac23-20	Apr. 19, 2023	Hogeun-dong, Seogwipo-si	M	43.1	256[a]	Pos
Ac23-15	Apr. 19, 2023	Hogeun-dong, Seogwipo-si	M	44.4	512[a]	Pos
Ac23-19	Apr. 19, 2023	Hogeun-dong, Seogwipo-si	M	32.6	128[a]	Pos
Ac22-24	Mar. 24, 2022	Bongseong-ri, Jeju-si	F	42.2	512[b]	Pos
Ac22-19	Mar. 24, 2022	Bongseong-ri, Jeju-si	M	33.9	32[a]	Pos
Ac23-1	Apr. 18, 2023	Sangdae-ri, Jeju-si	M	33.2	256[a]	Pos
Ac23-17	Apr. 19, 2023	Hogeun-dong, Seogwipo-si	M	35.2	1024[a]	Pos
Ac23-22	Apr. 19, 2023	Hogeun-dong, Seogwipo-si	F	34.9	256[a]	Pos
Ac23-12	Apr. 19, 2023	Hogeun-dong, Seogwipo-si	F	28.3	128[a]	Pos
Ac23-14	Apr. 19, 2023	Hogeun-dong, Seogwipo-si	F	27.4	Neg[a]	Pos
Ac22-20	Mar. 24, 2022	Bongseong-ri, Jeju-si	M	16.3	64[a]	Pos
Ac22-23	Mar. 24, 2022	Bongseong-ri, Jeju-si	F	26.4	512[b]	Pos

[a], Indirect immunofluorescence antibody (IFA) test was conducted on sera; [b], IFA test was conducted on heart fluids. IgG, immunoglobulin G; RT-PCR, reverse transcription-polymerase chain reaction; Ac, *Apodemus chejuensis*; M, male; F, female; Pos, positive; Neg, negative.
