# Supplementary material for: Phylogenetic diversity and molecular evolution of Hantaan virus harbored by Apodemus chejuensis on Jeju Island, Republic of Korea, 2022–2023
Source: PLoS Negl Trop Dis. 2025 Aug 19;19(8):e0013459. doi: 10.1371/journal.pntd.0013459 (PMC12373272; doi:10.1371/journal.pntd.0013459)
Supplement: S3 Table — (PDF) [file pntd.0013459.s005.pdf]

S3 Table. Hantaan virus RNA copy numbers from the lung tissues of *Apodemus chejuensis* collected on Jeju Island, Republic of Korea.

Viral RNA copy number (copies/uL)	Sample	Collection site		Ct value
10^5 to 10^6	Ac23-18	Seogwipo-si	Hogeun-dong	15.9
	Ac23-20	Seogwipo-si	Hogeun-dong	16.0
	Ac23-15	Seogwipo-si	Hogeun-dong	17.8
	Ac23-19	Seogwipo-si	Hogeun-dong	18.0
	Ac22-24	Jeju-si	Bongseong-ri	20.3
10^4 to 10^5	Ac23-1	Jeju-si	Sangdae-ri	23.0
	Ac23-17	Seogwipo-si	Hogeun-dong	23.7
	Ac22-19	Jeju-si	Bongseong-ri	24.2
10^2 to 10^3	Ac23-12	Seogwipo-si	Hogeun-dong	27.2
	Ac23-22	Seogwipo-si	Hogeun-dong	29.0
0 to 1	Ac23-14	Seogwipo-si	Hogeun-dong	n.d
	Ac22-20	Jeju-si	Bongseong-ri	n.d
	Ac22-23	Jeju-si	Bongseong-ri	n.d

Ct, cycle threshold; Ac, *Apodemus chejuensis*; n.d., not determined.
