# Supplementary material for: Phylogenetic diversity and molecular evolution of Hantaan virus harbored by Apodemus chejuensis on Jeju Island, Republic of Korea, 2022–2023
Source: PLoS Negl Trop Dis. 2025 Aug 19;19(8):e0013459. doi: 10.1371/journal.pntd.0013459 (PMC12373272; doi:10.1371/journal.pntd.0013459)
Supplement: S4 Table — (PDF) [file pntd.0013459.s006.pdf]

S4 Table. Summary of mapped reads and sequencing depth of amplicon-based nanopore sequencing for Hantaan virus harbored by *Apodemus chejuensis* collected on Jeju Island, Republic of Korea, in 2022–2023.

Viral RNA copy number (copies/µL)	Sample	Total reads	Reads mapped to reference / Total reads (%)	S segment		M segment		L segment	
				Reads mapped (%)	Depth of coverage[a]	Reads mapped (%)	Depth of coverage[a]	Reads mapped (%)	Depth of coverage[a]
10^5 to 10^6	Ac23-18	88,265	77,569 (87.9)	15,364	3,378	27,136	2,251	35,069	1,466
	Ac23-20	369,354	333,664 (90.3)	61,471	13,228	120,591	9,501	151,602	6,287
	Ac23-15	432,686	389,251 (90.0)	74,603	16,107	147,312	11,738	167,336	6,845
	Ac23-19	371,825	337,980 (90.9)	87,558	17,690	121,543	9,436	128,879	4,869
	Ac22-24	500,721	458,614 (91.6)	130,826	27,549	169,796	12,881	157,892	6,237
10^4 to 10^5	Ac23-1	696,741	640,475 (91.9)	238,116	47,653	74,429	4,852	327,930	12,802
	Ac23-17	393,961	356,980 (90.6)	115,157	22,020	120,486	8,529	121,337	4,505
	Ac22-19	281,405	252,649 (89.8)	74,123	47,653	80,571	4,852	97,955	12,802
10^2 to 10^3	Ac23-12	735,758	639,276 (87)	212,884	30,871	170,306	8,900	256,086	8,361
	Ac23-22	515,996	485,899 (94)	229,459	34,571	91,479	5,454	164,961	4,772
0 to 1	Ac23-14	539,211	343,038 (63.6)	313,687	48,566	26,016	1,526	3,335	128
	Ac22-23	109,577	2,073 (1.9)	934	241	495	42	644	29
	Ac22-20	124,044	13,334 (10.7)	1,597	1,598	1,929	124	10,355	42
**Average**		**396,888**	**333,139 (83.9)**	**119,675 (30.2)**	**23,933**	**88,622 (22.3)**	**6,160**	**124,875 (31.5)**	**5,319**

[a]; Sequencing depth was calculated as the number of mapped reads (read length × number of reads matching the reference/reference genome size). Ac, *Apodemus chejuensis*.
