# Supplementary material for: Phylogenetic diversity and molecular evolution of Hantaan virus harbored by Apodemus chejuensis on Jeju Island, Republic of Korea, 2022–2023
Source: PLoS Negl Trop Dis. 2025 Aug 19;19(8):e0013459. doi: 10.1371/journal.pntd.0013459 (PMC12373272; doi:10.1371/journal.pntd.0013459)
Supplement: S5 Table — (PDF) [file pntd.0013459.s007.pdf]

S5 Table. Nucleotide and amino acid sequence similarities of Hantaan virus (HTNV) carried by *Apodemus chejuensis* collected on Jeju Island, Republic of Korea, in 2022–2023.

	Percentage of nucleotide and amino acid sequence identities in HTNV (compared with reference genome sequence of HTNV Ac20-5)					
	S segment		M segment		L segment	
Sample	Nucleotide (%)	Amino acid (%)	Nucleotide (%)	Amino acid (%)	Nucleotide (%)	Amino acid (%)
Ac22-19	99.2	99.5	99.2	99.6	99.5	99.6
Ac22-24	99.2	99.4	99.1	99.5	99.5	99.5
Ac23-1	99.2	99.6	99.2	99.6	99.4	99.6
Ac23-15	99.2	99.6	99.2	99.6	99.5	99.6
Ac23-17	99.2	99.5	99.2	99.6	99.5	99.6
Ac23-18	99.2	99.5	99.2	99.6	99.5	99.6
Ac23-19	99.2	99.5	99.2	99.6	99.5	99.6
Ac23-20	99.2	99.6	99.2	99.6	99.5	99.6
Ac23-22	99.2	99.5	99.2	99.6	99.8	99.6

S, small; M, medium; L, large; Ac, *Apodemus chejuensis*.
