# Supplementary material for: Phylogenetic diversity and molecular evolution of Hantaan virus harbored by Apodemus chejuensis on Jeju Island, Republic of Korea, 2022–2023
Source: PLoS Negl Trop Dis. 2025 Aug 19;19(8):e0013459. doi: 10.1371/journal.pntd.0013459 (PMC12373272; doi:10.1371/journal.pntd.0013459)
Supplement: S6 Table — (PDF) [file pntd.0013459.s008.pdf]

S6 Table. Accession numbers of genomic sequences of Hantaan virus (HTNV) S, M, and L segments in the current study.

Strain	Year	Site	Nation	Accession No.		
				S segment	M segment	L segment
HTNV Ac22-19	2022	Jeju	ROK	PQ410874	PQ410883	PQ410892
HTNV Ac22-24	2022	Jeju	ROK	PQ410875	PQ410884	PQ410893
HTNV Ac23-1	2023	Jeju	ROK	PQ410876	PQ410885	PQ410894
HTNV Ac23-15	2023	Seogwipo	ROK	PQ410877	PQ410886	PQ410895
HTNV Ac23-17	2023	Seogwipo	ROK	PQ410878	PQ410887	PQ410896
HTNV Ac23-18	2023	Seogwipo	ROK	PQ410879	PQ410888	PQ410897
HTNV Ac23-19	2023	Seogwipo	ROK	PQ410880	PQ410889	PQ410898
HTNV Ac23-20	2023	Seogwipo	ROK	PQ410881	PQ410890	PQ410899
HTNV Ac23-22	2023	Seogwipo	ROK	PQ410882	PQ410891	PQ410900
HTNV Ac19-6	2019	Jeju	ROK	MW219768	MW219762	MW219756
HTNV Ac20-5	2020	Jeju	ROK	MW219769	MW219763	MW219757
HTNV Ac20-6	2020	Jeju	ROK	MW219770	MW219764	MW219758
HTNV Ac20-30	2020	Jeju	ROK	MW219771	MW219765	MW219759
HTNV Ac20-31	2020	Jeju	ROK	MW219760	MW219766	MW219772
HTNV Ac20-32	2020	Jeju	ROK	MW219773	MW219767	MW219761
HTNV CU Aa17-1	2017	Gwangju	ROK	OR113725	OR113716	OR113707
HTNV CU Aa17-2	2017	Gwangju	ROK	OR113735	OR113734	N.D[a]
HTNV CU Aa17-3	2017	Boseong	ROK	N.D[a]	N.D[a]	N.D[a]
HTNV CU Aa17-5	2017	Boseong	ROK	N.D[a]	N.D[a]	N.D[a]
HTNV CU Aa17-6	2017	Boseong	ROK	N.D[a]	N.D[a]	N.D[a]
HTNV CU Aa19-40	2019	Boseong	ROK	OR113726	OR113717	OR113708
HTNV CU Aa19-96	2019	Boseong	ROK	OR113727	OR113718	OR113709
HTNV CU Aa19-102	2019	Boseong	ROK	OR113728	OR113719	OR113710
HTNV CU Aa19-106	2019	Boseong	ROK	OR113729	OR113720	OR113711
HTNV CU Aa19-109	2019	Boseong	ROK	OR113730	OR113721	OR113712
CUH 2017-1155	2017	Unknown	ROK	OR113738	OR113737	OR113736
CUH 2018-603	2018	Unknown	ROK	OR113741	OR113740	OR113739
CUH 2020-003	2020	Unknown	ROK	OR113731	OR113722	OR113713
CUH 2020-509	2020	Unknown	ROK	OR113732	OR113723	OR113714
CUH 2021-1167	2021	Unknown	ROK	OR113733	OR113724	OR113715
CUH 2021-1188	2021	Unknown	ROK	OR113744	OR113743	OR113742
HTNV Aa03-387	2003	Yeoncheon	ROK	KT935026	KT934992	KT934958
HTNV Aa05-331	2005	Yeoncheon	ROK	KT935030	KT934996	KT934962
HTNV Aa05-190	2005	Paju	ROK	KT935027	KT934993	KT934959
HTNV Aa14-172	2014	Paju	ROK	KT935042	KT935008	KT934974
HTNV Aa09-410	2009	Pocheon	ROK	KU207193	KU207185	KU207177

HTNV Aa09-948	2009	Pocheon	ROK	KT935034	KT935000	KT934966
HTNV Aa04-722	2004	Pocheon	ROK	KU207190	KU207182	KU207174
HTNV Aa15-82	2015	Hwacheon	ROK	MT012548	MT012560	MT012572
HTNV Aa15-84	2015	Hwacheon	ROK	MT012549	MT012561	MT012573
HTNV Aa17-337	2017	Cheorwon	ROK	MT012550	MT012562	MT012574
HTNV Aa17-353	2017	Cheorwon	ROK	MT012551	MT012563	MT012575
HTNV Aa17-367	2017	Cheorwon	ROK	MT012552	MT012564	MT012576
HTNV Aa17-421	2017	Chuncheon	ROK	MT012553	MT012565	MT012577
HTNV Aa17-422	2017	Chuncheon	ROK	MT012554	MT012566	MT012578
HTNV Aa18-164	2018	Yanggu	ROK	MT012555	MT012567	MT012579
HTNV Aa18-179	2018	Yanggu	ROK	MT012556	MT012568	MT012580
HTNV Aa18-185	2018	Yanggu	ROK	MT012557	MT012569	MT012581
HTNV 76-118	1976	Dongducheon	ROK	M14626	NC005219	NC005222
HTNV 84FLi	2001	Shaanxi	China	AF366568	AF345636	AF336826
HTNV A9	2000	Jiangsu	China	AF329390	AF035831	AF293665
HTNV HV004	2012	Hubei	China	JQ083395	JQ083394	JQ083393
HTNV Q32	2006	Guizhou	China	AB027097	DQ371905	DQ371906
HTNV TJJ16	2003	Tianjin	China	AY839871	EU074672	KU215675
HTNV Z10	2018	Zhejiang	China	NC_006433	NC_006437	NC_006435

[a] Genomic sequences were kindly provided by Prof. Won-Keun Kim at Hallym University, Chuncheon, ROK.

ROK, Republic of Korea; N.D, No data.
